# Dose-response relationship of taurine on endurance cycling performance under hot and humid conditions

**DOI:** 10.3389/fnut.2025.1632131

**Published:** 2025-10-13

**Authors:** Xinlei Li, Lei Huo, Linuo Wang, Wenjuan Zhang

**Affiliations:** ^1^Department of Physical Education, Henan Normal University, Xinxiang, Henan, China; ^2^Zhengzhou Tourism College, Zhengzhou, China; ^3^Department of Physical Education, Xi’an University of Finance and Economics, Xi’an, China; ^4^Department of Military Theory, Fuzhou University, Fuzhou, China

**Keywords:** taurine, endurance cycling performance, hot and humid condition, dosage, thermoregulatory responses

## Abstract

**Purpose:**

To investigate the effects of different doses of taurine on endurance exercise performance and physiological parameters under hot and humid conditions.

**Methods:**

This study adopted a double-blind, randomized, placebo-controlled, cross-over design. Sixteen male university students majoring in physical education (age: 20.12 ± 1.12 years; training status: ≥3 endurance sessions/week) received 4 supplement conditions: (1) placebo (maltodextrin), (2) low-dose (1 g taurine), (3) medium-dose (4 g taurine), and (4) high-dose (6 g taurine). Participants performed a graded cycling test (initial 50 W, +50 W every 3 min at 70 rpm) until exhaustion in an environmental chamber (35 °C, 65% RH). Heart rate, core temperature, skin temperature, sweat rate, RPE, and thermal sensation were measured.

**Results:**

Compared to placebo, time to exhaustion was significantly longer in the medium-dose group (*p* < 0.05), with no significant or trend-level effects in low- or high-dose groups. Blood lactate accumulation and sweating rate were higher in the medium-dose group (*p* < 0.05). Core temperature (9 min-End) was lower in the medium-dose group during the latter exercise phase (*p* < 0.05).

**Conclusion:**

Under hot and humid conditions, acute 4 g taurine supplementation enhanced time to exhaustion during graded cycling by improving thermoregulatory responses, whereas 1 g and 6 g doses showed no ergogenic effects.

## 1 Introduction

Exercise performance in hot and humid environments is significantly impaired due to reduced evaporative cooling efficiency (1), leading to accelerated (2) strain and thermoregulatory failure (3, 4). While proper hydration strategies can partially mitigate these effects, targeted nutritional interventions such as taurine supplementation may offer additional protection by addressing underlying physiological stressors (5). Taurine, a conditionally essential amino acid, has demonstrated particular relevance for thermal stress management through three primary mechanisms (6): its antioxidant capacity to scavenge exercise-induced reactive oxygen species (7), osmoregulatory function in maintaining cellular volume and electrolyte balance during dehydration (8), and direct thermoregulatory effects through enhanced sweat gland responsiveness and hypothalamic regulation (9–11). These distinct mechanisms position taurine as a uniquely promising ergogenic aid for humid heat conditions exceeding 60% relative humidity (6, 12).

Current understanding of taurine’s dose-response relationship remains unclear due to conflicting evidence across studies. While low-dose acute supplementation (1 g) showed no performance benefits in cycling time trials (13), chronic high-dose administration (6 g/day) improved maximal oxygen uptake (7) but required prolonged use (14). Animal studies suggest moderate doses (3–4 g) may offer acute thermoregulatory advantages, though human data under controlled environmental stress remain limited (6, 10, 15). This inconsistency likely stems from variations in exercise protocols, environmental conditions, and supplementation regimens, highlighting the need for systematic dose comparisons under standardized heat stress conditions (6).

Emerging evidence specifically suggests taurine’s efficacy in hot environments may differ from its general ergogenic effects. Studies have demonstrated that acute taurine intake can increase sweating rates by 12.7% at 35 °C (16), while chronic supplementation appears to delay critical core temperature thresholds during progressive heat exposure (12). However, existing research has predominantly examined these effects at moderate humidity levels (≤40% RH) (6, 16), leaving a critical gap in understanding taurine’s potential under the more physiologically challenging conditions of high humidity (≥60% RH) where evaporative cooling is substantially impaired.

Therefore, the present study aimed to investigate the acute effects of three doses of taurine (1, 4, and 6 g) on endurance cycling performance and thermoregulatory responses under controlled hot and humid conditions (35 °C and 65% relative humidity). We hypothesized that moderate-dose taurine (4 g) would optimally enhance time to exhaustion by improving thermoregulatory efficiency, while lower and higher doses would prove less effective due to insufficient physiological stimulation and potential osmotic stress, respectively (13, 17–19). This investigation provides the first systematic evaluation of taurine’s dose-dependent ergogenic effects under conditions of combined heat and high humidity, offering practical insights for athletic performance in environmentally challenging conditions.

## 2 Methods

### 2.1 Research design

This study adopted a double-blind, randomized, placebo-controlled, cross-over design. Participants reported to the laboratory in 5 visits. Participants familiarized with the experimental equipment and procedures and adjusted the power bike seat and handles position during their 1st visit to the lab.

During the formal experiment (2–5 visits), participants were divided into a placebo group (P), a low-dose group (L), a medium-dose group (M), and a high-dose group (H) according to the random assignment method (GraphPad, https://www.graphpad.com/quickcalcs/randomize1.cfm).

A seven-day interval between conditions was selected to allow for complete recuperation from the protocols and sufficient time to consume the crossover supplementation. Taurine has a clearance/bioavailability ratio of approximately 21 h, which was deemed an adequate washout period (17, 20). The entire experiment was conducted within a Controlled High-Temperature and High-Humidity Chamber, meticulously regulated by a combination of heating (SAWO, CON4, Finland) and humidifying (BELIN, SC-G060ZS, CHN) equipment, ensuring a precise temperature range of 35 °C ± 1 °C and humidity level of 65% ± 2%, thereby fostering optimal conditions for the experimental proceedings. This study received institutional ethical approval (Capital University of Physical Education and Sports, No. 2020A55) and was performed in conformity with the 1964 Helsinki Declaration.

### 2.2 Procedure

**Figure 1:**
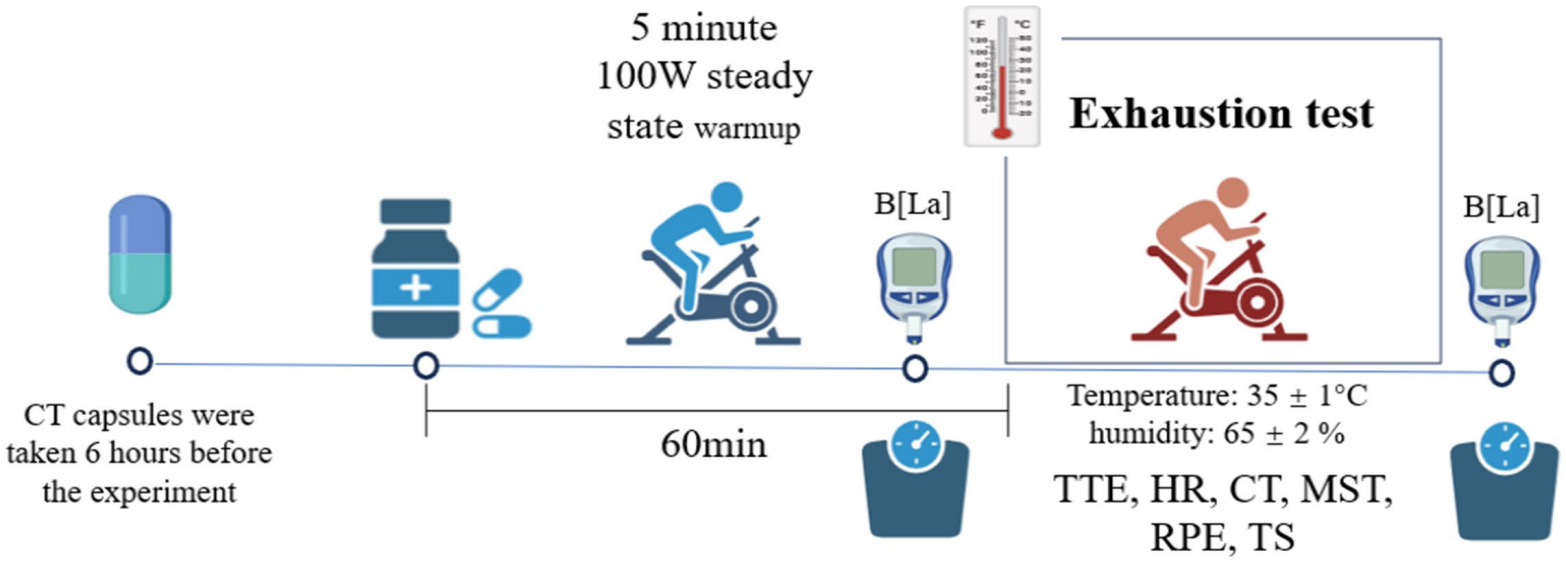
Visualization of experimental procedure flow.

Participants took a temperature capsule to measure gastrointestinal temperature 6 h before performing the exhaustion test to ensure that the temperature capsule was not in the stomach and to avoid the temperature capsule being affected by ingested food or beverages. Participants ingested 300 mL of water mixed with supplements 1 h before the start of the test to ensure a homogeneous status. Before warming up, participants put on a heart rate belt and skin temperature buttons. Participants were initially performed in a laboratory environment (25 °C, 30% RH) for a 5 min 100 W steady-state warm-up. Subsequently, the body weight was measured. Then, the participants entered a high-temperature and humidity chamber for an exhaustion test. Heart rate, core temperature, skin temperature, RPE, and thermal sensation were recorded throughout the exhaustion test. Exhaustion time was recorded immediately after the exhaustion test. Then, the body was dried with a towel and the weight was measured again (Figure 1).

### 2.3 Participants

Sixteen male college students majoring in physical education volunteered to participate in this study (age: 20.12 ± 1.12 years; stature: 1.76 ± 0.06 m; body mass: 72.06 ± 5.99 kg; maximal oxygen uptake (V̇_O2max_): 46.24 ± 4.07 mL/kg/min). *A priori* sample sizes were calculated using G*Power (Version No. 3.1.9.7. Franz Faul University Kiel, Germany) (21). The parameters are set as follows: *α* = 0.05, power = 0.85, effect size = 0.25. Participants voluntarily enrolled in this experiment and were aware of the procedure and requirements and the potential for uncomfortable reactions. The Physical Activity Readiness Questionnaire (PAR-Q) was used to identify those at risk for exercise. The experimental protocol, though involving thermal stress, was conducted under controlled conditions with predefined termination criteria to ensure safety. Participants signed an informed consent form and ensured that they had sufficient time to complete the experiment. The inclusion criteria were as follows: (1) 1. Aged between 18 and 22 years old, with at least 3 physical training sessions per week; (2) Good health, no major illnesses, no injuries or surgeries within the last 6 months; (3) No intake of any ergogenic aid in the last 3 months; and (4) No habit of exercising in high temperature or high humidity environments.

### 2.4 Supplementation and standardization

Prior research has classified taurine intake doses into three categories: low (0.5–2 g), medium (3–5 g), and high (>5 g) (6). All supplements were prepared in powder form and measured using an analytical balance and subsequently ingested in liquid form, mixed with 300 mL of water. Participants ingested the supplement 1 hour prior to the experiment. 1 h timing was chosen as this accounted for the peak plasma availability of taurine after oral administration. Participants were randomized to the following 4 supplementation groups: placebo (maltodextrin), low-dose (1 g TAU), medium-dose (4 g TAU), and high-dose (6 g TAU), followed by a randomized crossover trial (22). Before the experiment, participants were asked to record their diets. They were then instructed to repeat these diets 24 h before the formal experiment. Throughout the study period, participants were prohibited from consuming any nutritional supplements. Compliance was verified through daily dietary logs, periodic interviews, and random spot checks (with participant consent). Any supplement use would have resulted in immediate exclusion from the study. All subjects entered the laboratory at least 3 h after eating. The same period in 1 day was selected to complete each experiment to minimize the effects of circadian rhythms (8:00 a.m.–10:00 a.m.).

### 2.5 Measurements

#### 2.5.1 Time to exhaustion

TTE is frequently employed as a measure of endurance performance in a laboratory setting (23). The participants started cycling (LODE 906900, Netherlands) at 50 W and increased 50 W every three (24).

Participants were asked to maintain a pedal cadence of 70 rpm until complete exhaustion. Exhaustion was defined as voluntary withdrawal or a decrease in pedal cadence to below 60 rpm for more than 10 s. The TTE is recorded immediately at the end of the exhaustion test.

#### 2.5.2 Heart rate

Participants wore a polar heart rate belt throughout the session (Polar H10, Finland). The heart rate belt must be in close contact with the skin, paying particular attention to the wearing position at the middle connection between the chest and the abdomen.

#### 2.5.3 Blood lactate

BLa was obtained by testing ear blood with a blood lactate meter (EKF Diagnostics Holdings plc, Cardiff, United Kingdom). BLa was measured before and immediately after the exhaustion test.

#### 2.5.4 Sweat rate

SR was calculated by collecting information on the participant’s body weight before and after the exercise session and using the following formula: Sweat rate = (Δmass + fluid)/exercise time (4, 25).

#### 2.5.5 Core temperature

Core temperature was measured using an ingestible, noninvasive temperature capsule (e-CELSIUS^®^, BMedical Pty LTb, Australia), which was taken by the subject 6 h before the experiment (26). The temperature capsule was used to measure human gastrointestinal temperature (gastrointestinal temperature), and the sampling frequency of the temperature capsule was set to 30 s. After connecting with an external sensor (e-CELSIUS^®^, BMedical Pty LTb, Australia), the core temperature data were received and the gastrointestinal temperature was continuously monitored.

#### 2.5.6 Mean skin temperature

Skin temperature was measured using temperature recording buttons (DS1922L, Maxim Integrated, United States), which were attached to the sternal incision, forearm, thigh, and calf to record skin temperatures of the chest (T_chest_), forearm (Tarm), thigh (T_thigh_), and calf (T_calf_). The whole body MST was calculated using the formula: MST = 0.3 (Tchest + Tarm) + 0.2 (Tthigh + Tcalf) (4).

#### 2.5.7 Rating of perceived exertion

RPE was recorded on a 6 to 20-point Borg scale (27). RPE was recorded at baseline, every 3 min after the start of the exhaustion test, and immediately after the end of the exhaustion test. Participants provided verbal ratings, which were immediately documented by the research team.

#### 2.5.8 Thermal sensation

TS was recorded on a 9-point scale where −4 = “very cold,”0 = “neutral,” and 4 = “very hot.” TS was recorded using the same verbal recording method as RPE.

### 2.6 Statistical analysis

The statistical analyzes were conducted using the SPSS version 27.0 software package (IBM Corp, Armonk, NY, United States). The data is presented as mean ± standard deviation (SD). The normality test was assessed by Shapiro–Wilk for all the data and the sphericity test was performed on the data by Mauchly test. CT, B[La], HR, TS, and RPE were analyzed using repeated measures of two-way (group x time) ANOVA. TTE and SR were analyzed using a one-way ANOVA with Bonferroni correction. The statistical significance level was set at *p* < 0.05. Effect sizes were interpreted using Cohen’s guidelines for partial η^2^ (0.01 = small, 0.06 = medium, 0.14 = large).

## 3 Results

### 3.1 Time to exhaustion

**Figure 2:**
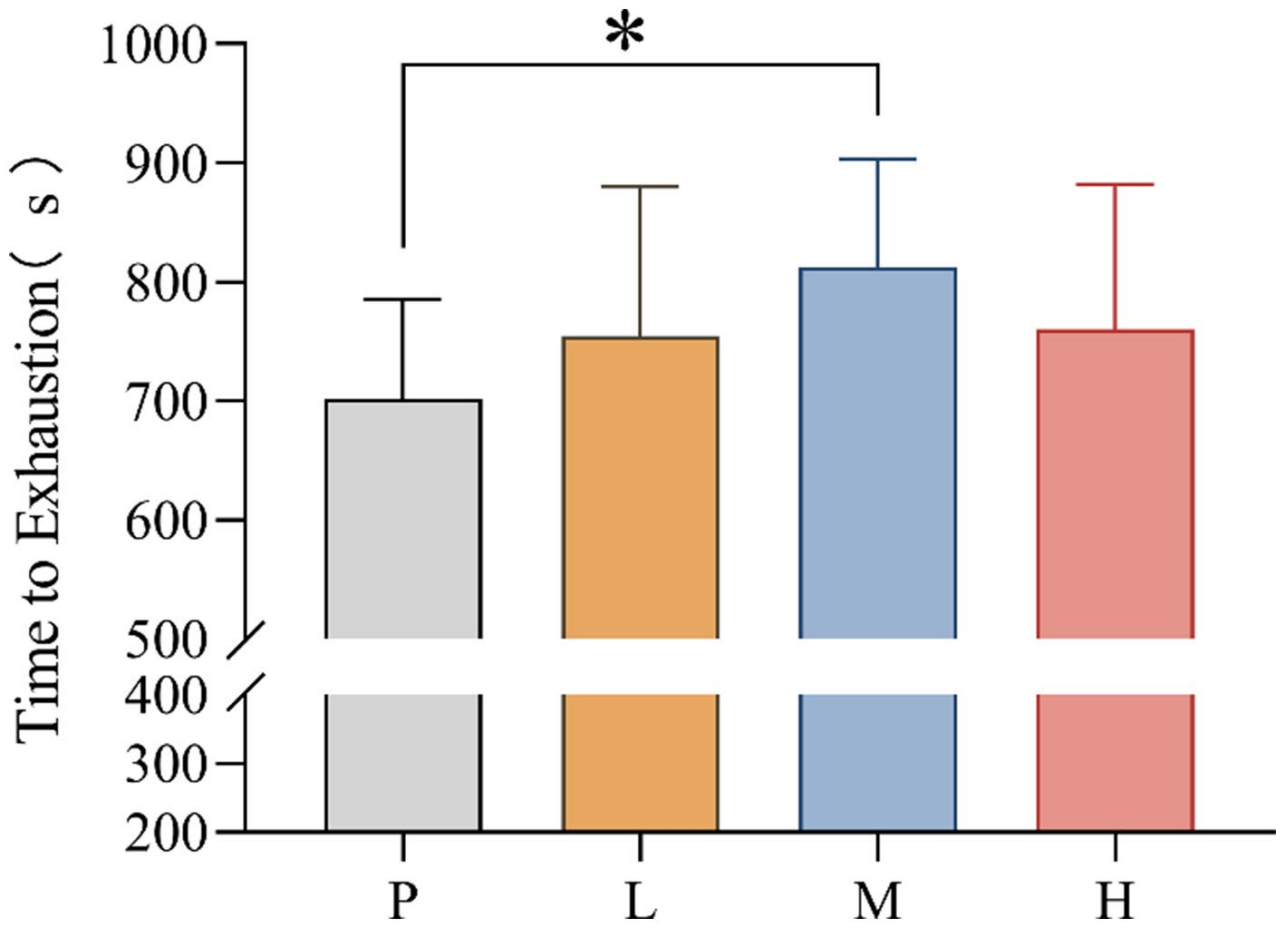
Time to exhaustion (* *p* < 0.05) after supplementation with different doses of taurine or placebo (*n* = 16) under hot and humid conditions (35 °C, 65% RH).

TTE values for P (mean = 702.38; SD = 83.088), L (mean = 754.81, SD = 125.378), M (mean = 812.38, SD = 90.948) and H (mean = 760.44, SD = 121.962) groups were recorded. The data showed significant differences in TTE between interventions, *F*_(3, 33)_ = 4.858, MSE = 1442.365; *p* = 0.046. Follow-up multiple comparisons showed that TTE was significantly higher in the M group than in the P group (*p* = 0.031) (Figure 2).

### 3.2 Heart rate

**Figure 3:**
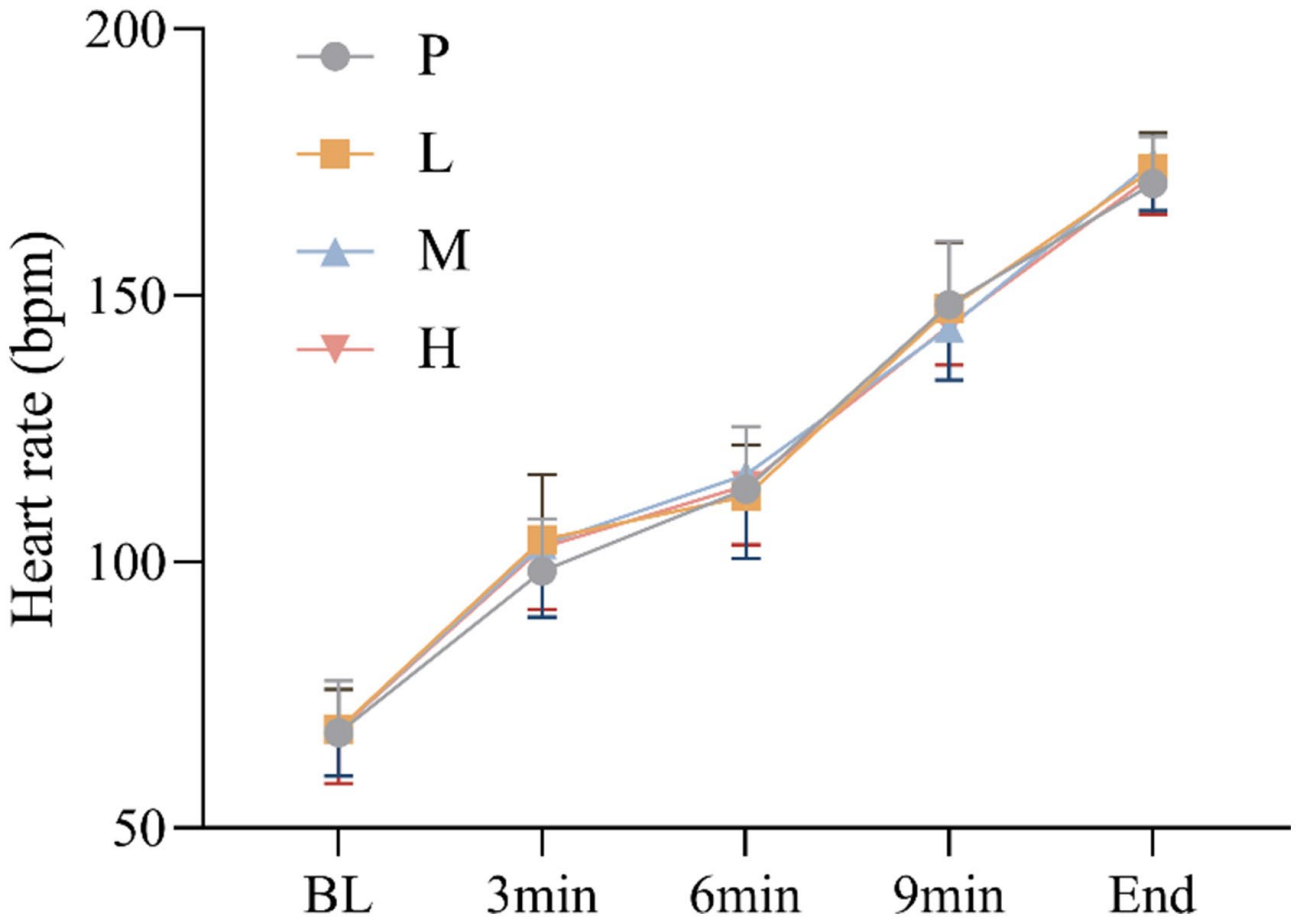
Changes in HR during the exhaustion test under hot and humid conditions (35 °C, 65% RH).

These results showed no significant main effect of intervention [*F*_(3, 60)_ = 0.237, *p* = 0.870, η^2^_partial_ = 0.012], and a significant main effect of time, Greenhouse–Geisser adjusted [*F*_(4, 57)_ = 1177.885, *p* < 0.001, η^2^_partial_ = 0.988], there was no significant intervention × time interaction effect, Greenhouse–Geisser adjusted [*F*_(4, 59)_ = 2.018, *p* = 0.104, η^2^_partial_ = 0.120] (Figure 3).

### 3.3 Blood lactate

**Figure 4:**
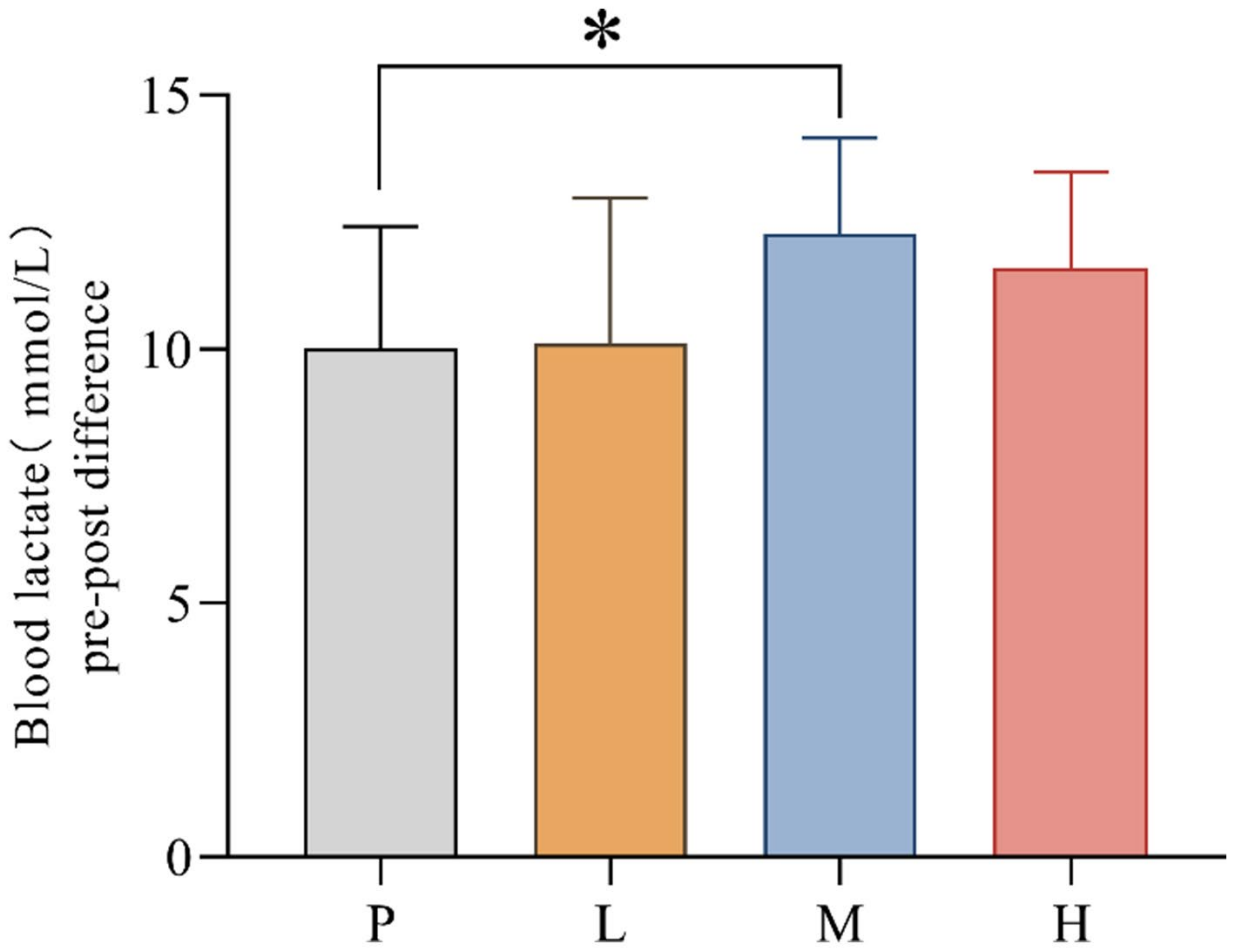
Comparison of blood lactate differences pre- and post-exhaustion testing (* *p* < 0.05) in 4 groups under hot and humid conditions (35 °C, 65% RH).

B[La] pre-post difference values for P (mean = 10.006; SD = 2.406), L (mean = 10.130, SD = 2.879), M (mean = 12.275, SD = 1.882) and H (mean = 11.600, SD = 1.892) groups were recorded. The data showed significant differences in B[La] pre-post difference between interventions, *F*_(3, 33)_ = 3.876, MSE = 5.30; *p* = 0.014. Follow-up multiple comparisons showed that B[La] pre-post difference was significantly higher in the M group than in the P group (*p* = 0.043) (Figure 4).

### 3.4 Sweat rate

**Figure 5:**
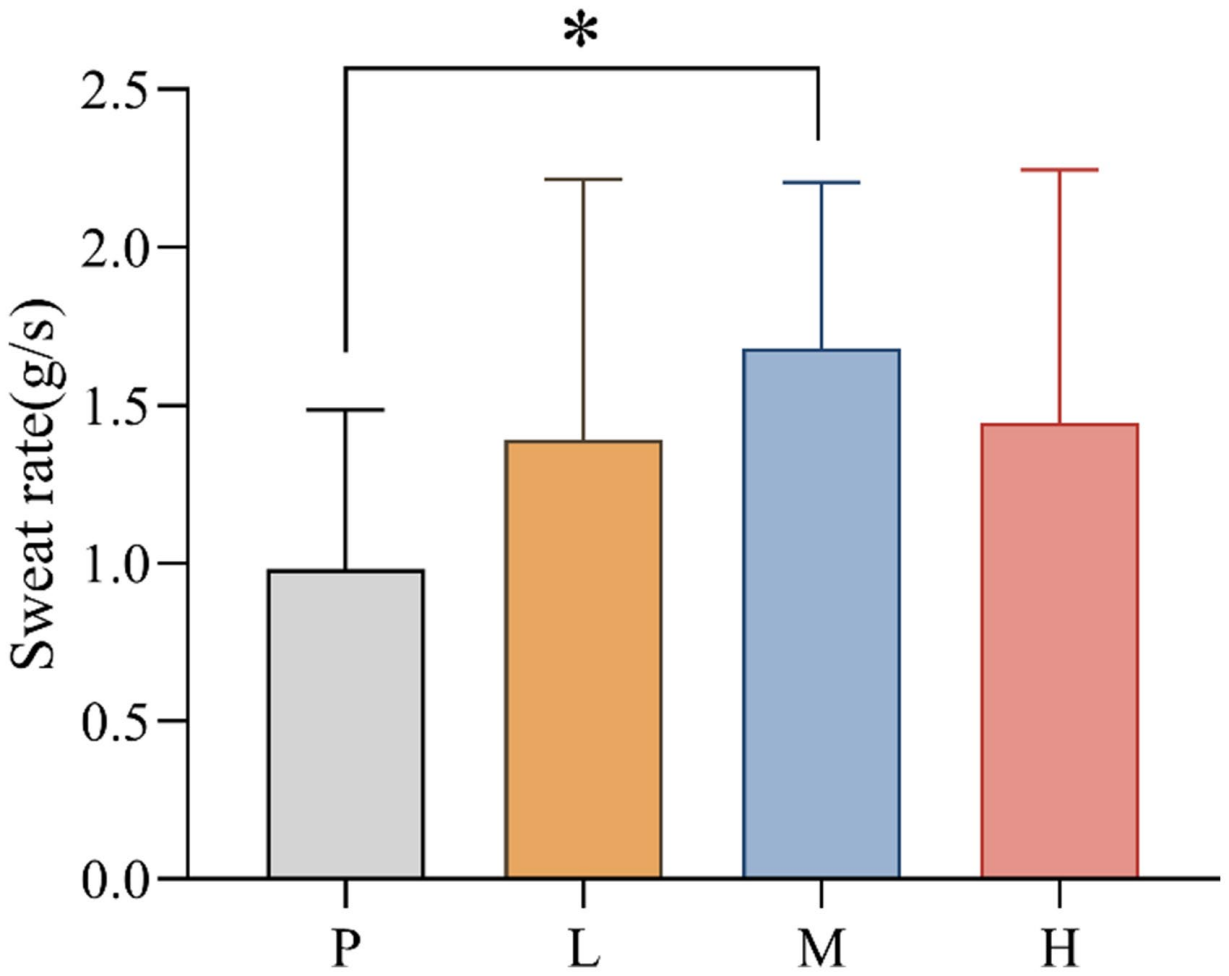
Comparison of SR (* *p* < 0.05) in 4 groups under hot and humid conditions (35 °C, 65% RH).

SR values for P (mean = 0.981; SD = 0.506), L (mean = 1.388, SD = 0.828), M (mean = 1.681, SD = 0.525) and H (mean = 1.444, SD = 0.802) groups were recorded. The data showed significant differences in SR between interventions, *F*_(3, 33)_ = 4.858, MSE = 0.465; *p* = 0.041. Follow-up multiple comparisons showed that SR was significantly higher in the M group than in the P group (*p* = 0.031) (Figure 5).

### 3.5 Core temperature

**Figure 6:**
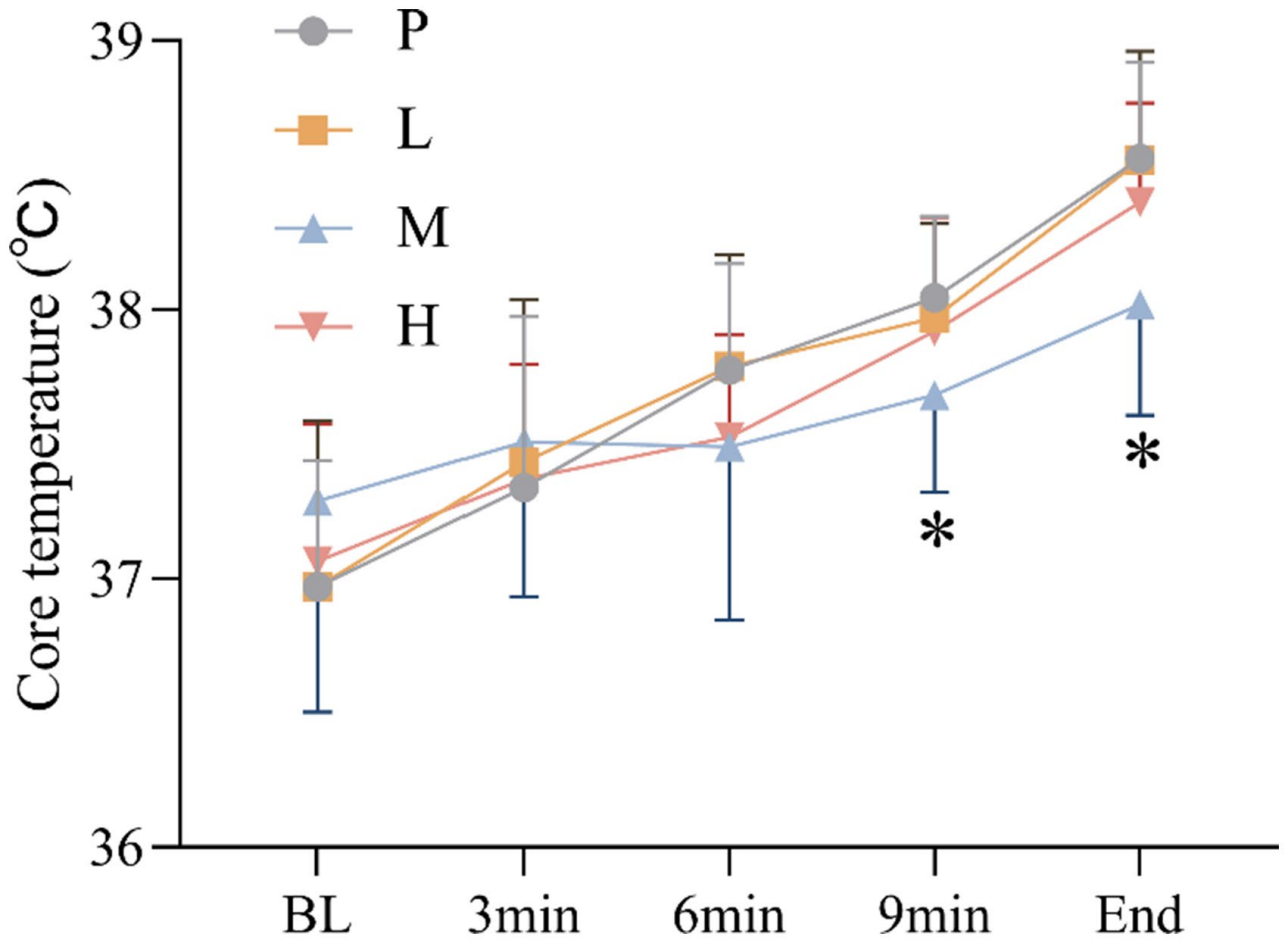
Changes in CT (* *p* < 0.05) during the exhaustion test under hot and humid conditions (35 °C, 65% RH).

These results showed no significant main effect of intervention [*F*_(3, 60)_ = 1.051, *p* = 0.377, η^2^_partial_ = 0.050], and a significant main effect of time, Greenhouse–Geisser adjusted [*F*_(4, 57)_ = 49.150, *p* < 0.001, η^2^_partial_ = 0.775], there was significant intervention × time interaction effect, Greenhouse–Geisser adjusted [*F*_(4, 59)_ = 3.689, *p* = 0.010, η^2^_partial_ = 0.200]. CT was significantly lower in group M than in group P at 9 min (*p* = 0.037) and at the end of exhaustion (*p* = 0.001) (Figure 6).

### 3.6 Mean skin temperature

**Figure 7:**
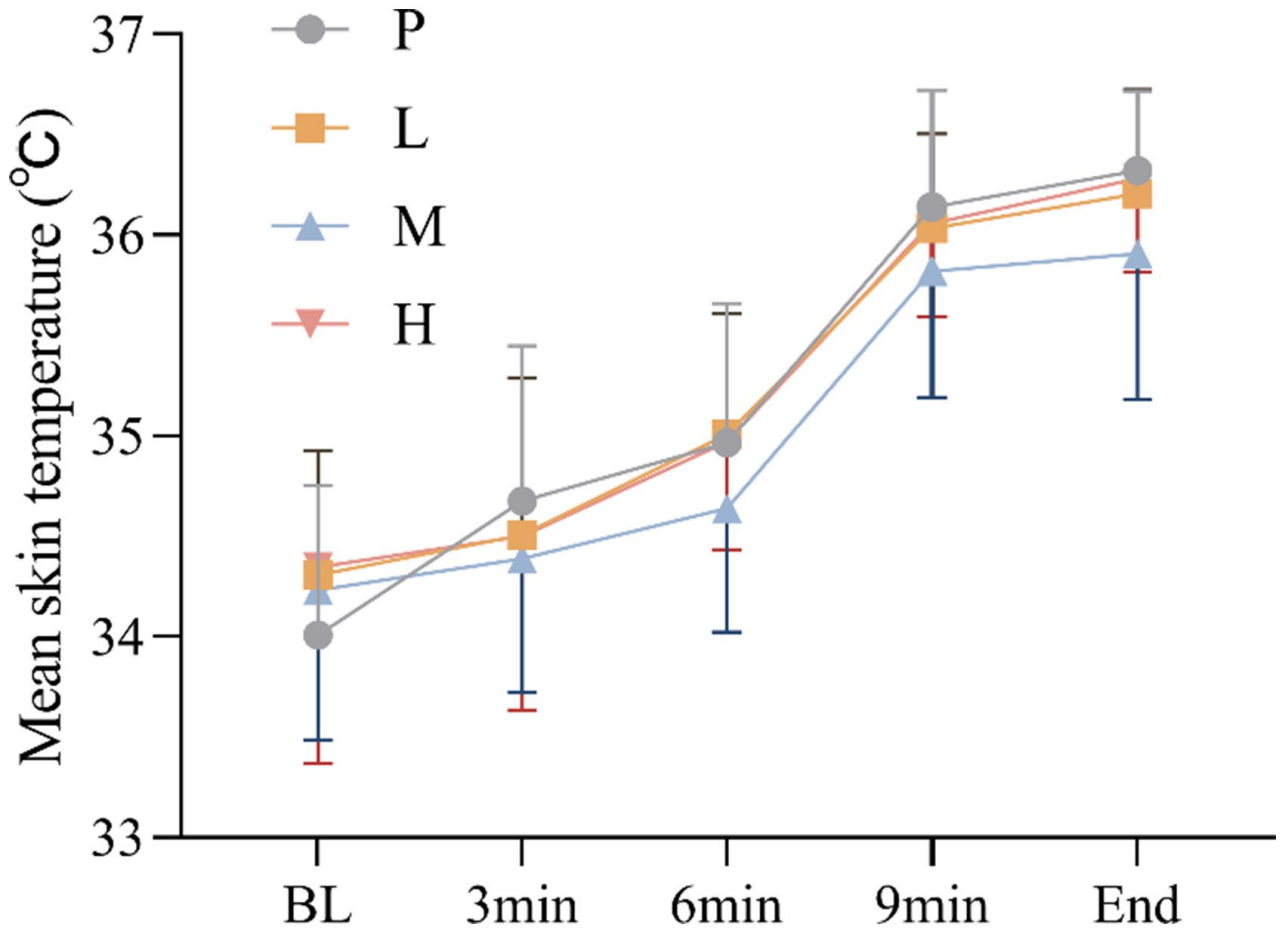
Changes in MST during the exhaustion test under hot and humid conditions (35 °C, 65% RH).

These results showed no significant main effect of intervention [*F*_(3, 60)_ = 1.964, *p* = 0.129, η^2^_partial_ = 0.089], and a significant main effect of time, Greenhouse–Geisser adjusted [*F*_(4, 57)_ = 94.695, p<0.001, η^2^_partial_ = 0.869], there was no significant intervention × time interaction effect, Greenhouse–Geisser adjusted [*F*_(4, 59)_ = 1.276, *p* = 0.290, η^2^_partial_ = 0.080] (Figure 7).

### 3.7 Rating of perceived exertion and thermal sensation

For RPE, these results showed no significant main effect of intervention [*F*_(3, 60)_ = 0.300, *p* = 0.826, η^2^_partial_ = 0.015], and a significant main effect of time, Greenhouse–Geisser adjusted [*F*_(4, 57)_ = 366.065, *p* < 0.001, η^2^_partial_ = 0.963], there was no significant intervention × time interaction effect, Greenhouse–Geisser adjusted [*F*_(4, 59)_ = 2.093, *p* = 0.093, η^2^_partial_ = 0.124].

**Figure 8:**
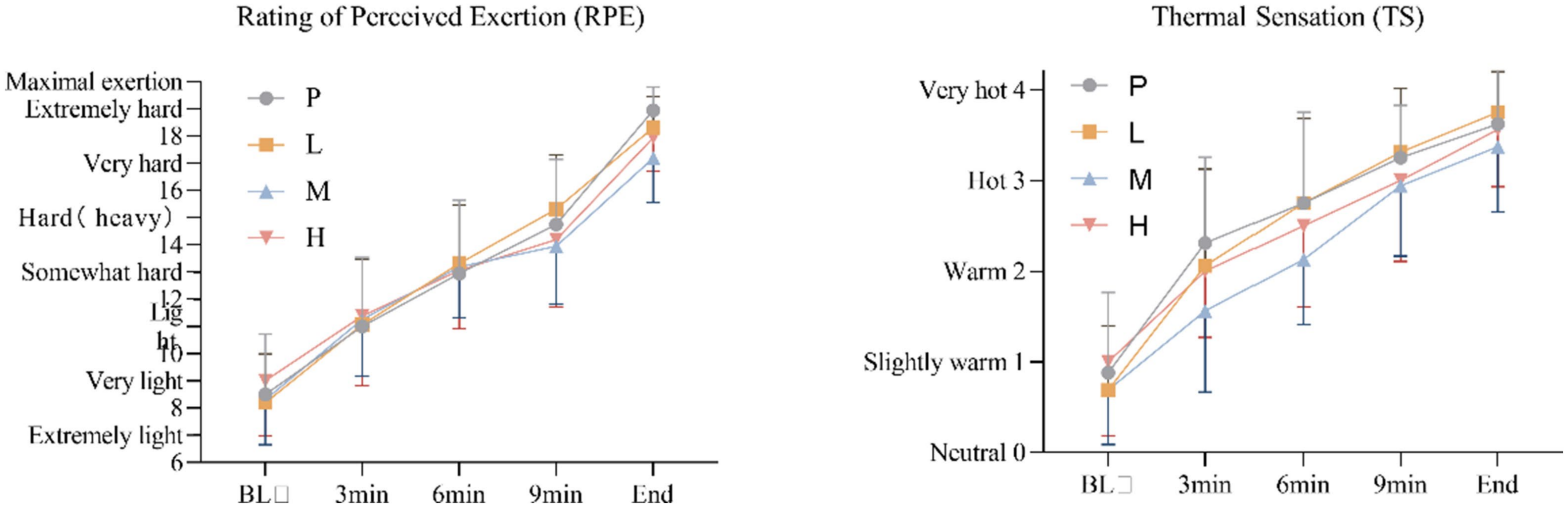
Changes in RPE and TS during the exhaustion test under hot and humid conditions (35 °C, 65% RH).

For TS, these results showed no significant main effect of intervention [*F*_(3, 60)_ = 1.867, *p* = 0.145, η^2^_partial_ = 0.085], and a significant main effect of time, Greenhouse–Geisser adjusted [*F*_(4, 57)_ = 151.966, *p* < 0.001, η^2^_partial_ = 0.914], there was no significant intervention × time interaction effect, Greenhouse–Geisser adjusted [*F*_(4, 59)_ = 1.631, *p* = 0.178, η^2^_partial_ = 0.100] (Figure 8).

## 4 Discussion

The present study investigated the dose-response effects of acute taurine supplementation on endurance cycling performance under hot and humid conditions (35 °C, 65% RH). Our findings demonstrate that moderate-dose taurine (4 g) significantly improved time to exhaustion by 12.4% compared to placebo, while low (1 g) and high (6 g) doses showed no ergogenic benefits. These results suggest a U-shaped dose-response relationship, with 4 g emerging as the optimal dose for performance enhancement in these environmental conditions.

The performance benefits observed with 4 g taurine appear mediated through improved thermoregulation. Specifically, we found that this dose reduced core temperature during the latter stages of exercise while increasing the sweat rate. These findings align with previous work showing taurine’s ability to enhance evaporative cooling through sweat gland modulation (16) and hypothalamic thermoregulation (28). The higher sweat rate may reflect taurine’s role in upregulating aquaporin-5 expression in eccrine sweat glands (6, 29), though future studies should directly measure this mechanism. Importantly, the humid conditions in our study (65% RH) likely amplified the thermoregulatory challenge, making these improvements particularly meaningful for athletic performance.

The lack of benefit from the 6 g dose warrants careful consideration. Several factors may explain this finding: First, high-dose taurine may induce osmotic diuresis that counteracts its thermoregulatory benefits (30). Second, intestinal taurine transporters may become saturated above 4 g, limiting bioavailability (31). Third, the combination of high dose and extreme humidity may have overwhelmed the body’s cooling capacity (14). While our data cannot definitively distinguish these possibilities, they highlight the importance of dose optimization for hot/humid conditions.

Metabolically, the 4 g group showed greater blood lactate accumulation post-exercise compared to placebo. This likely reflects their prolonged exercise duration rather than impaired clearance, as taurine typically reduces lactate accumulation in thermoneutral conditions (12, 16). The humidity-induced metabolic shift toward anaerobic glycolysis may explain this discrepancy (4, 10, 11), suggesting environmental conditions significantly modify taurine’s metabolic effects.

While we observed trends toward improved thermal sensation with 4 g taurine, subjective measures (RPE, TS) did not reach statistical significance. This may reflect the high interindividual variability in perceptual responses to heat stress (17, 18, 32), suggesting future studies should employ larger samples to detect potential psychological benefits.

Several limitations should be acknowledged. First, the lack of plasma taurine measurements prevents definitive conclusions about absorption and kinetics. Second, our graded exercise protocol differs from real-world endurance events, potentially limiting generalizability. Third, the homogeneous sample (young male athletes) necessitates caution when extrapolating to other populations. Future research should investigate weight-adjusted dosing (e.g., 50 mg/kg), include female and masters athletes, and examine prolonged exercise scenarios.

In conclusion, this study establishes that 4 g represents the optimal acute taurine dose for improving endurance performance in hot/humid conditions, primarily through enhanced thermoregulation. The U-shaped dose-response relationship underscores the importance of avoiding both insufficient and excessive dosing. These findings provide practical guidance for athletes and coaches while highlighting the need for further research on individualization strategies and long-term supplementation protocols.

## 5 Conclusion

Under hot and humid conditions, acute oral administration of a moderate 4 g taurine dose effectively enhances endurance exercise performance by improving thermoregulatory responses. This finding highlights the practical potential of taurine supplementation as a strategy to mitigate heat-related performance decrements. Future studies should explore dose-response relationships, utilizing plasma taurine levels to optimize dosing strategies for athletic and occupational populations exposed to extreme environments.

## Data Availability

The raw data supporting the conclusions of this article will be made available by the authors, without undue reservation.
